# Characterization of Graphite Oxide and Reduced Graphene Oxide Obtained from Different Graphite Precursors and Oxidized by Different Methods Using Raman Spectroscopy Statistical Analysis

**DOI:** 10.3390/ma14040769

**Published:** 2021-02-06

**Authors:** Roksana Muzyka, Sabina Drewniak, Tadeusz Pustelny, Marcin Sajdak, Łukasz Drewniak

**Affiliations:** 1 Institute for Chemical Processing of Coal, 1 Zamkowa St., 41-803 Zabrze, Poland; rmuzyka@ichpw.pl (R.M.); msajdak@ichpw.pl (M.S.); 2 Department of Optoelectronics, Faculty of Electrical Engineering, Silesian University of Technology, 2 Krzywoustego St., 44-100 Gliwice, Poland; tadeusz.pustelny@polsl.pl (T.P.); lukasz.drewniak@polsl.pl (Ł.D.)

**Keywords:** graphite, graphite oxide, reduced graphene oxide, Raman spectroscopy, XRD, XPS, HCA, ANOVA, Hummers method, Tour method

## Abstract

In this paper, various graphite oxide (GO) and reduced graphene oxide (rGO) preparation methods are analyzed. The obtained materials differed in their properties, including (among others) their oxygen contents. The chemical and structural properties of graphite, graphite oxides, and reduced graphene oxides were previously investigated using Raman spectroscopy (RS), X-ray photoelectron spectroscopy (XPS), and X-ray diffraction (XRD). In this paper, hierarchical clustering analysis (HCA) and analysis of variance (ANOVA) were used to trace the directions of changes of the selected parameters relative to a preparation method of such oxides. We showed that the oxidation methods affected the physicochemical properties of the final products. The aim of the research was the statistical analysis of the selected properties in order to use this information to design graphene oxide materials with properties relevant for specific applications (i.e., in gas sensors).

## 1. Introduction

Graphene, obtained in 2004 [1], is characterized by its extraordinary physical and chemical properties [2]. A worldwide effort has been initiated to develop efficient methods of producing graphene and its derivatives. The most widely used chemical production method for graphene is the oxidation of graphite and the exfoliation–reduction of the obtained graphite oxide. The basic preparation methods were developed by Brodie [3], Staudenmaier [4], Hoffmann [5], Hummers [6], Hummers and Offemann [7], and Tour [8,9]. The last two methods (Hummers–Offemann [6] and Tour [8,9] methods) deserve special attention. Hummers and Offemann presented an optimized, rapid, safe method of graphite oxidation, whereby GO is obtained via treatment of graphite by strong oxidizing agents. This method is currently the most used. Meanwhile, Tour et al. [9] suggested the partial replacement of nitric acid by less corrosive phosphoric acid. This limits the generation of toxic gases.

The literature reports that the efficiency of the oxidation processes and the resulting GO structure depend on the type of graphite and its structural parameters—mainly the size of the crystallites [10,11]. The obtained graphite oxide is a multilayer structure. Its color depends on the origin and size of the graphite grains and degree of oxidation (whereby the color changes from dark grey to green-blue) [10,11,12,13]. The obtained graphite oxides contain oxygen in their structures in amounts ranging from 25% to 60% by mass, and the materials contain different structures, compositions, and ratios of carbon to oxygen (C/O). The influence of the oxidation method on the oxygen content in the GO is shown in Table 1.

Based on the literature [14,15,16], we can hypothesize a relationship between the graphite precursor and the composition, morphology, and structure of the obtained graphite oxides. Moreover, the used oxidation method also affects the structure of the graphite oxides. In turn, the literature [17] shows that the origin and structure of the graphite and its oxidation method significantly impact the composition, distribution of oxygen connections, and structure of the obtained GO. The degree of comminution of the graphite is an additional factor that affects the effectiveness of the oxidation process. Deemer et al. [18] studied the influence of the oxidation method (using the Hummers [6] and Marcano–Tour [9] methods) using various sizes (smaller than 100 µm and bigger than 420 µm) of graphite flake. In the Hummers method, the graphene materials’ parameters depend on the grain size, which is correlated with the oxidation degree of GO. In [19], it is shown that the size of the crystallites, oxidation degree, and types of oxygen connections play important roles in creating composites of GO with metals. Along with increases in the oxygen content, carboxylic groups located on the edges of the graphene flakes take part in the creation of the composites. Epoxy and hydroxyl groups form bonds with metals only when the carboxylic acid content in the GO structure is very low.

The graphene oxide is obtained through reduction and exfoliation. This process can be performed in many ways, one of which seems to be promising for the mass production of graphene materials—the so-called "thermal method" [20,21].

Our researchers [14,22,23] confirmed the dependence of the type of graphite precursor on the properties of reduced graphene oxides. The influence of three graphite precursors was studied, i.e., natural flake graphite (GF), natural scaly graphite (GS), and synthetic graphite (GE). The highest degree of oxidation of graphite was achieved using the modified Hummers and Tour methods. Moreover, we observed that larger graphite oxides were obtained with larger crystallite dimensions in the graphite, irrespective of the oxidation method. A distinct dependence of the degree of reduction on the graphite precursor was also observed. The highest degree of reduction and smallest number of structural defects were obtained for reduced graphene oxide prepared using flake graphite (rGO-F), whose crystallite diameter was the largest among the examined graphite materials. The rGO obtained from scalar graphite showed the smallest degree of reduction and share of carbon with C sp^2^ hybridization and the highest degree of structural defects. It is also worth mentioning that the largest surface area values (over 900 m^2^g^−1^) were measured for reduced graphene oxides obtained from scale and flake graphites and oxidized using a modified version of Tour’s method [9].

The mentioned papers and our research [16,24], based on RS, SEM, XPS, and XRD measurements, show that both the graphite precursor and the oxidation method determine the properties of the fabricated GO and rGO.

For this purpose, it is possible to apply chemometric approaches, which have been successfully used not only for analysis of the possible relationship between the graphite precursor and oxidation method, but also for many other applications, such as for the design of polymeric sensor materials (which materials make it difficult to predict the desired properties) [25,26,27]. Another good example related to our study is the use of the chemometric methods for the design of biological sensors, e.g., based on molecularly imprinted polymers (MIP). The chemometrics methods can be applied to investigate the effects of variables such as the type and quantity of monomers, cross-linkers, porogens, initiators, types of initiation (UV or thermal), polymerization pressure, temperature, reaction time, and reaction vial dimensions on the properties of synthesized polymers [28,29]. Chemometric methods can be used to study the relationships between the variables under investigation and for optimization of the sensor properties [25].

This paper aims to show how the graphite precursors and graphite oxide oxidation methods determine the physicochemical properties of graphene oxides (which were fabricated using different methods derived from the Hummers method (A, B, C) and a modified version of the Tour method (D). The presented analyses can help in selecting a fabrication technology for graphite oxide to obtain a material with specific physicochemical properties (for a specific practical application).

## 2. Materials and Methods

### 2.1. The Selection of Graphite for the Preparation of Graphite Oxide and Reduced Graphene Oxide

In order to obtain graphite oxides and reduced graphene oxides, two natural flake (GF) and scalar (GS) graphites and one synthetic (GE) graphite were used. Each graphite (1 g) was ground and sieved to smaller than 20 μm, and in the next step was oxidized using one of four oxidation methods (A, B, C, or D; see Table 2). The names of the obtained graphite oxides follow the pattern of GOX-Y, where X is the type of graphite (S-scalar, F-flake, or E-synthetic) and Y is the type of oxidation method (A, B, C, or D). The reduction and exfoliation method was the same (thermal reduction) for each sample. Graphite oxides were annealed at 900 °C under a nitrogen flow of 50 mL/min. The samples were annealed at the final temperature for 5 min. It should also be mentioned that methods A, B, and C are versions of the Hummers [6] method, while method D is a modification of the Tour method [8].

### 2.2. Methods of Characterization for the Obtained GO and rGO

The following characterization methods were used for the graphite oxides and graphene oxides: X-ray diffraction (XRD), X-ray photoelectron microscopy (XPS), Raman spectroscopy (RS), and elementary analysis.

For XRD measurements, the samples were deposited onto glass and analyzed using Cu Ka1 radiation, with a voltage of 45 kV and a current of 30 mA. In the experiments, an X’Pert PRO PW 3040/60 diffractometer (PANalytical, Quebec, Canada) was used. (exemplary spectra are shown in SI files, Figures S4 and S5).

In the XPS measurements, a PHI 500 VersaProbe spectrometer (Chigasaki, Japan) was used (Al Kα anode radiation beam: 1486.6 eV).

The Raman spectra were recorded using an N-TEGRA Spectra platform (NT-MDT, Eindhoven, the Netherlands). In the measurements, a laser beam with a 532 nm wavelength was used (the exposure time was 10 s). (Exemplary spectra are shown in SI files, Figures S2 and S3).

The elementary analysis results were obtained using a Vario Macro Cube automatic elementary analyzer (Elementar Analysysteme GmbH Company, Hanau, Germany).

### 2.3. Data Analysis—Chemometric Approach

One of the commonly used methods for exploratory multivariate data is clustering analysis (CA). CA is one of the chemometric methods used to describe the relationships among different variables. CA allows the grouping of samples characterized by significant similarity (e.g., chemical similarity) and allows the identification of outliers. This method is often used in chemical analyses and environmental studies for the following reasons:

- To distinguish between sources of emissions [30];
- To identify areas with different levels of pollution [31];
- For the analysis of solid fuels, e.g., coal [32];
- To determine the physicochemical properties of the pollution fractions in the environment [33].

The goal of CA is to combine tested samples or variables into clusters that indicate mutual similarities or differences. This study uses the hierarchical clustering analysis (HCA) method, in which groups are built gradually, starting with single observations that are then combined into larger clusters. Splitting of clusters, starting from the first cluster (cluster covering all objects), is performed by successive division of the first cluster into smaller, more homogeneous clusters.

The first step in the analysis is to calculate the distance between features in the multidimensional data space, e.g., via Euclidean distance (as was done in the discussed studies). This allows the similarity between the tested objects or variables to be assessed, assuming that similar objects are adjacent.

For this purpose, the raw data were subjected to a standardization process to obtain a data set characterized by a distribution with a mean equal to “0” and standard deviation equal to “1”. Dataset standardization is an essential stage in data preprocessing, especially in the case of variables characterized by different units and different data values, e.g., 0.0001 to 100.00. The differences between these data would make it impossible to compare the tested variables through HCA analysis. The next step in conducting HCA is to determine the rules for joining clusters, i.e., determining when two clusters are similar enough to be able to combine them. In the presented research, we applied the complete method, which is also called furthest neighbor grouping. The grouping results can be visualized as a dendrogram and clustered heat maps can show cluster merger sequences and the distances at which each merger took place. In the presented studies, the complete linkage method was applied.

To determine the statistically significant impact of the graphene precursors’ studied properties and the used methods, analysis of variance (ANOVA) was applied. ANOVA helps to indicate the variables that affect the quality of the GO and rGO, e.g., the oxygen concentration, C/O ratio, amounts of Csp^2^ and Csp^3^ bands, interlayer distance d_001_, and average size of the crystallites L_a_.

This approach indicates which variables are statistically significant (e.g., V1, V2, and V3, where V is a variable) and the interactions among these variables (e.g., V1V2, V1V3, V2V3, and V1V2V3). To achieve this goal, all experimental data were statistically analyzed using MATLAB (MATLAB, 2016).

## 3. Results

**Figure 1:**
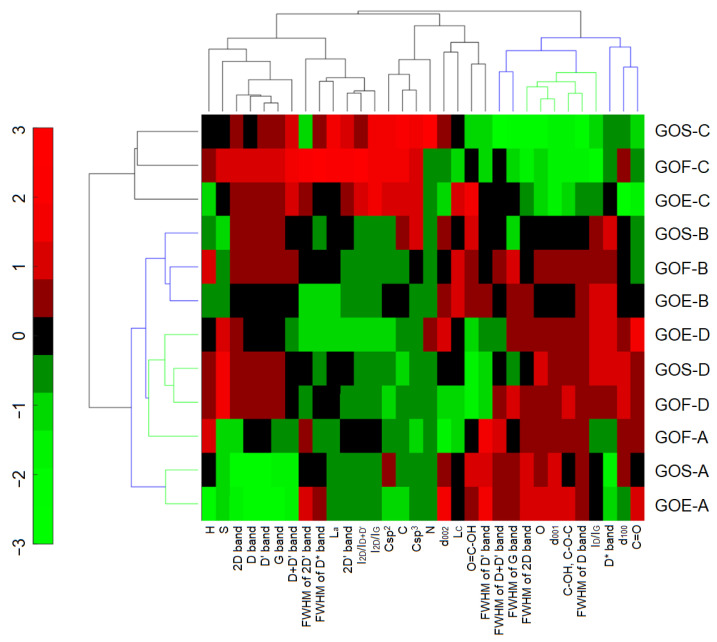
The clustered heat map for the studied GO samples and their properties.

The efficiency of the oxidation processes for the three graphites with four oxidation methods was 140–156% by mass. In this case, the efficiency is expressed as a percentage and determines the increase in the mass of tested graphite samples caused by the inclusion of oxygen atoms in the graphite structure during the oxidation process. The highest efficiency was obtained by applying method D, while the lowest efficiency was obtained by applying method C. This efficiency directly translated into the following oxygen contents: method C < method B < method D < method A (up to 36%). This trend is shown in Figure 1 (dendrograms marked in blue) as a result of HCA. The GOs produced using methods D and A (Figure 1, dendrogram marked in green) were characterized by high and homogeneous results of several parameters, such as the oxygen contents, interlayer distances d_001_, and contents of epoxy and hydroxyl groups.

As mentioned earlier, cluster analysis makes it possible to determine the similarity between the study samples and the variables used to describe their characteristics. In the clustered heat map (Figure 1), the similarity scale is in the range of <−3.3> and is a consequence of the performed normalization of all variables (without which a comparative analysis of the measured variables would be impossible; a schematic presentation of the normalization of all variables is presented in Figure S1). The interpretation of the presented graphs in the form of a clustered heat map can be compared to linear regression and proportionality. If the tested samples or variables have comparable similarity values, e.g., −3, it can be concluded that the relationship between them is directly proportional. An example is the oxygen content in samples oxidized by method C. The data obtained using elemental analysis (the changes of oxygen content) are directly proportional to the data obtained using XPS spectroscopy (oxygen content: C=O, C–O–H, C–O–C, O=C–OH embedded in the tested material). A similar interpretation is performed in the case of extreme values, e.g., −3, 3. For example, such similarity values for oxygen contents characterize samples obtained using methods A and C, whereby method A was characterized by the highest mean oxygen value of 35% (similarity value of 3) and method C was characterized by the lowest mean oxygen value of 22% (similarity value of −3). As can also be seen based on CA analysis and in terms of oxygen content, methods A, D, and B can be linked into one group (this is also confirmed by the results; for these three methods graphite oxides are characterized by oxygen contents higher than 30%.

**Figure 2:**
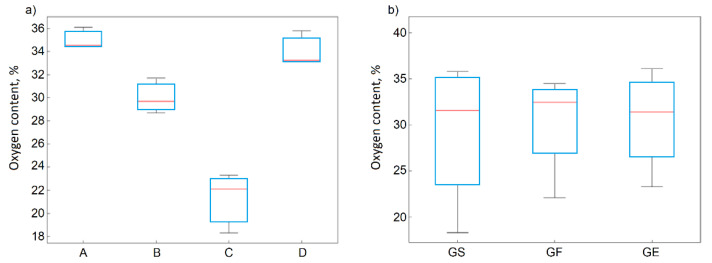
Box plots for oxygen concentrations depending on the (**a**) method and (**b**) precursor material.

The statistical data analyses and the ANOVA method confirmed the significant statistical influence of the oxidation method on the oxygen content (Figure 2a) for the GO, in contrast to the kind of graphite precursor (Figure 2b). It also confirmed that the oxygen content depended on the oxidation method in the following order: method C < method B < method D < method A.

**Figure 3:**
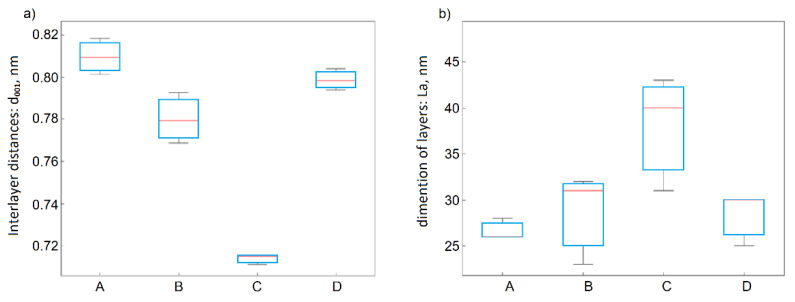
Box plots for (**a**) d_001_ and (**b**) L_a_ as functions of the applied method.

During the graphite oxidation process, the interlayer distances between the graphene layers increased more than two-fold, from approximately 0.336 nm (band 002) to 0.8184 nm (band 001), as a result of adding oxygen groups between layers and adding oxygen groups at the edges of the layers. The graphite oxides obtained using method A were characterized by the largest d_001_ interlayer distances (0.8013–0.8184 nm) and the smallest dimensions of graphene layers L_a_ (26–28 nm). The statistical significance of this information was confirmed by ANOVA, and the results for both d_001_ and L_a_ are presented in Figure 3a,b, respectively. It was observed that the larger dimensions of the graphene layers in the graphite, the larger dimensions were in the obtained GO, regardless of the oxidation method.

**Figure 4:**
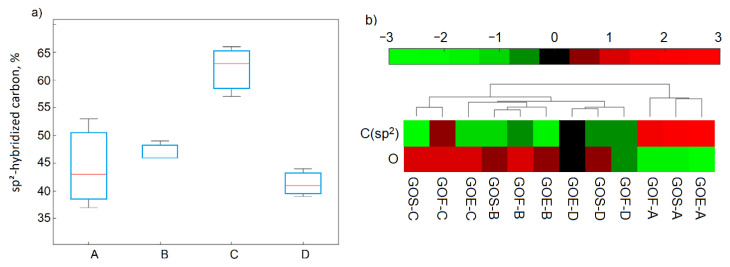
Box plot of C1sp^2^ hybridization as a function of the applied method (**a**) and clustered heat map of selected variables (for the studied GO) (**b**).

The deconvolution of XPS C1s spectra of graphite oxides showed that oxygen occurred mainly (i.e., more than 50% of the total oxygen content) in the epoxy and hydroxyl connections located on the graphite planes (at the edges there were fewer carbonyl and quinone or carboxylic groups). The total amounts of carbon with sp^2^ hybridization varied to a great extent in the graphite oxides (37–6%), which depended on the oxidation methods (Figure 4a) in a significant way. The carbon content with sp^2^ hybridization was inversely proportional to the oxidation yield, as shown in Figure 4b. The carbon content with this hybridization method increased when the degree of oxidation decreased and had the following trend: method C < method B < method A,D.

**Figure 5:**
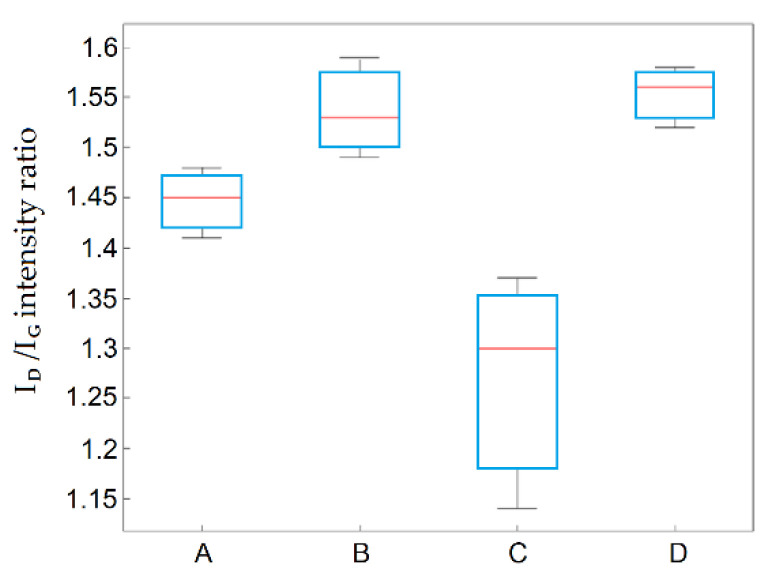
Diagram of between-group variation for I_D_/I_G_ as a function of the applied method.

The Raman spectroscopy confirmed an increase in the share of the disordered phase compared to that for the graphites, as evidenced by the increase of the D-band’s intensity in relation to the G-band. In the Raman spectra of the graphite oxides, both the G- and D-bands increased in width and shifted towards lower wavelengths, indicating an increase in the degree of defective structures due to the introduction of oxygen functional groups. The lowest I_D_/I_G_ intensity ratio was obtained for graphite oxides prepared using method C, which indicated that this method had the weakest oxidizing potential. The difference between graphite oxides obtained using method C and the others was statistically significant (the highest variability for the examined parameters was obtained for this method), as shown in Figure 5. The influence of the graphite precursor type (GF, GS, or GE) was statistically insignificant.

**Figure 6:**
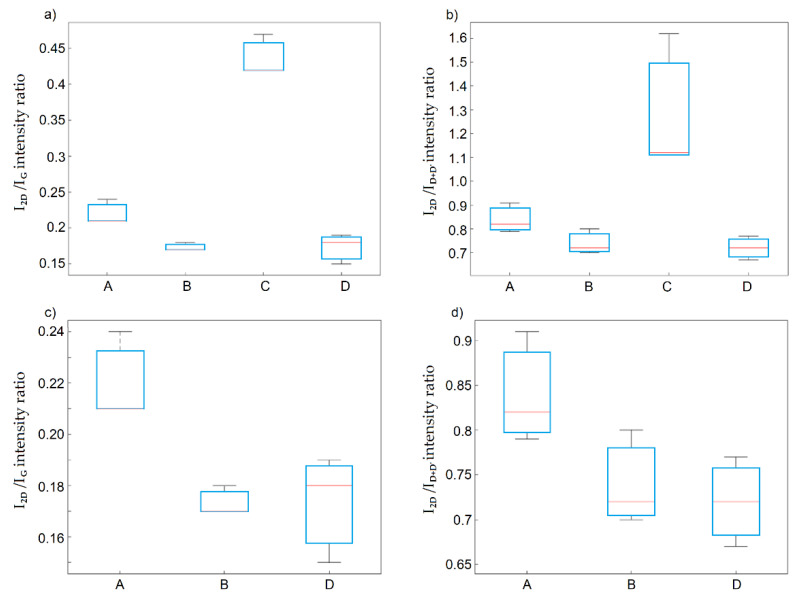
Box plots of: (**a**) I_2D_/I_G_ band ratio; (**b**) I_2D_/I_D+D’_ band ratio as a function of the applied method; (**c**) I_2D_/I_G_ band ratio; (**d**) I_2D_/I_D+D’_ after excluding method C.

The deconvolution of the 2D band and intensity ratios I_2D_ to I_G_ (I_2D_/I_G_) and I_2D_ to I_D+D’_ (I_2D_/I_D+D’_) showed that graphite oxides obtained using method D were composed of packets with the lowest numbers of graphene layers and the highest degrees of defective structures compared to those oxides obtained with the other methods. The differences in the I_2D_/I_G_ ratios of the graphite oxides obtained using method D were statistically significant in relation to methods A and C, while the I_2D_/I_D+D’_ intensity ratio for graphite oxides obtained using method D was statistically significant only in relation to method C (Figure 6). The largest number of graphite layers in the package was characterized for graphite oxides obtained using method C.

**Figure 7:**
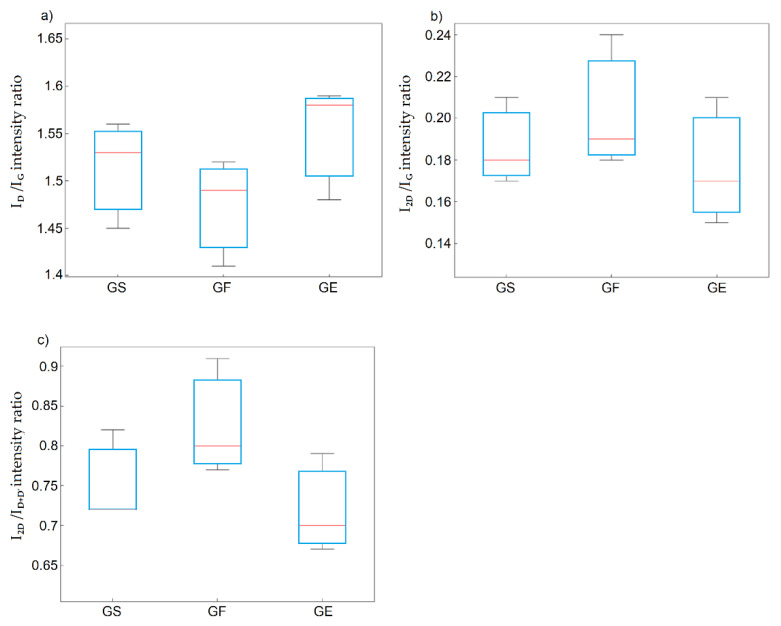
Box plots of: (**a**) I_D_/I_G_; (**b**) I_2D_/I_G_; (**c**) I_2D_/I_D+D’_ ratio as a function of the precursor material.

Analysis of data divided according to the type of graphite precursor may show that the highest I_D_/I_G_ ratio values and the lowest I_2D_/I_G_ and I_2D_/I_D+D’_ ratio values were obtained for graphite oxides containing synthetic graphite. This indicates that synthetic graphite was the most susceptible to the generation of structural defects and delamination of the graphite layers during the oxidation process. However, as shown from the statistical analysis (ANOVA method), these differences were statistically insignificant (Figure 7a–c).

**Figure 8:**
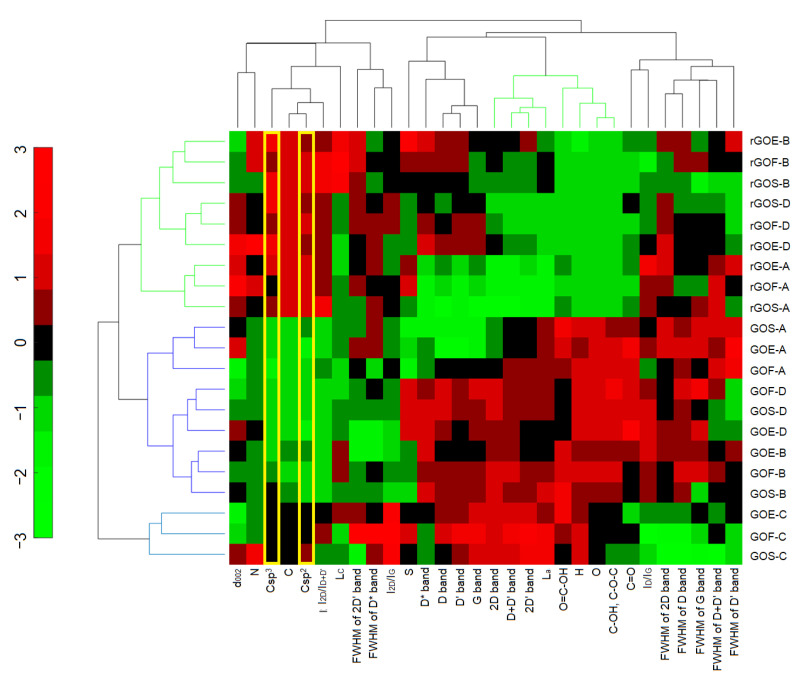
The clustered heat map for the studied GO and rGO samples and their properties.

The graphites oxidized using methods A, B, and D were selected to prepare reduced graphene oxides. The obtained graphene materials were characterized by 2–3-fold lower oxygen content than those for the corresponding graphite oxides. These differences were clearly visible when the collected data were analyzed using HCA. Figure 8 shows green dendrograms, which describe samples of reduced graphene oxides and variables characterizing the degree of oxidation of the investigated materials.

**Figure 9:**
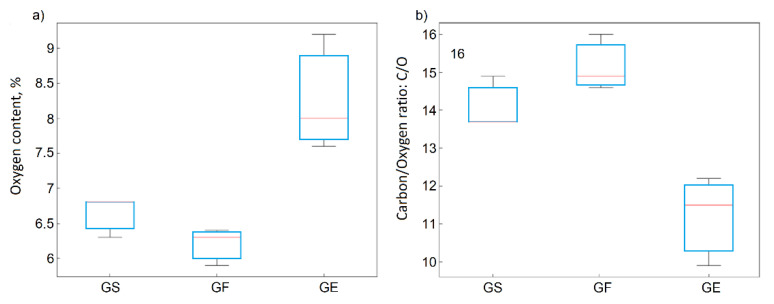
Box plots for the oxygen concentration (**a**) and carbon/oxygen ratios (**b**) depending on the precursor material.

The oxygen contents determined by XPS ranged from 6.3% to 9.2% at and varied depending on the type of graphite in the following order: rGO-F < rGO-S < rGO-E. The degree of the reduction represented by the C/O ratio increased in the following order: rGO-E < rGO-S < rGO-F. For both parameters mentioned above, the influence of the type of precursor was confirmed by ANOVA (Figure 9) and was statistically significant, thus confirming the observations made earlier.

The XPS C1s spectra analysis showed that oxygen in reduced graphene oxides (similar to GO) occurred mainly in the form of epoxy and hydroxyl groups and in smaller amounts in the form of carbonyl and quinone groups. Carboxylic groups are completely decomposed in the process of thermal exfoliation–reduction, while lactone groups appear. Graphite oxides and reduced graphene oxides are essentially different in the content of carbon with sp^2^ hybridization. The increase of C in sp^2^ hybridization from 37% to 53% for graphite oxides to 70% to 80% for reduced graphene oxides confirms that the graphite structure was partial reconstructed. These differences are visible in the results obtained during data analysis using the cluster method (Figure 8). The significant similarity for the number of carbon atoms with sp^2^ and sp^3^ bonds in rGO (high value of similarity ~2) compared to the number of sp^2^ and sp^3^ bonds in GO, for which the similarity value ranged from 0 to −3 (marked with yellow rectangles), is easy to notice.

**Figure 10:**
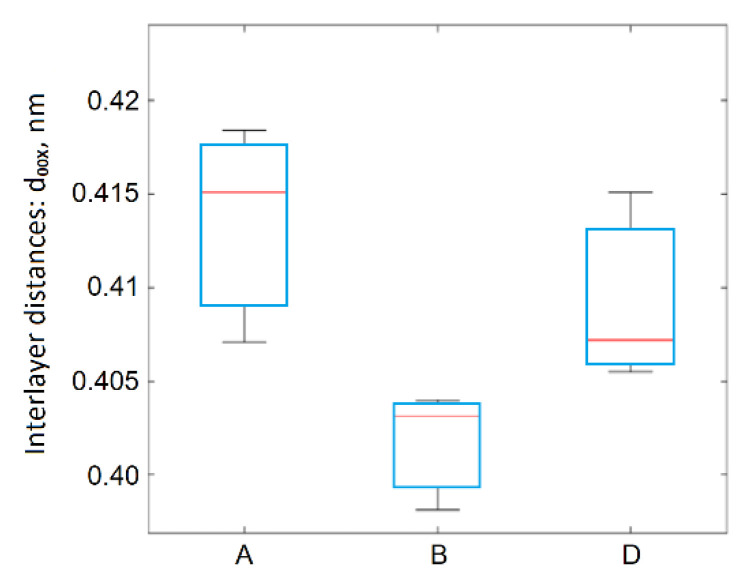
Diagram of between-group variation for d_00X_.

XRD studies confirmed the graphite structure reconstruction in the rGO. Interlayer distances decreased from ~0.8 nm (d_001_ band) in the GO to ~0.4 nm (d_00X_ band) in the rGO (Figure 10). The oxidation method’s influence affected the interlayer distances in the rGO, and this influence was statistically significant. Based on observations, it can be affirmed that reduced graphene oxides obtained from graphites oxidized by method A showed the highest degree of structural defects, while those obtained by method B were characterized by the lowest degree of structural defects. Such a low degree (for reduced graphene oxides prepared using method B) indicates that reconstruction of the graphite structure was increased.

**Figure 11:**
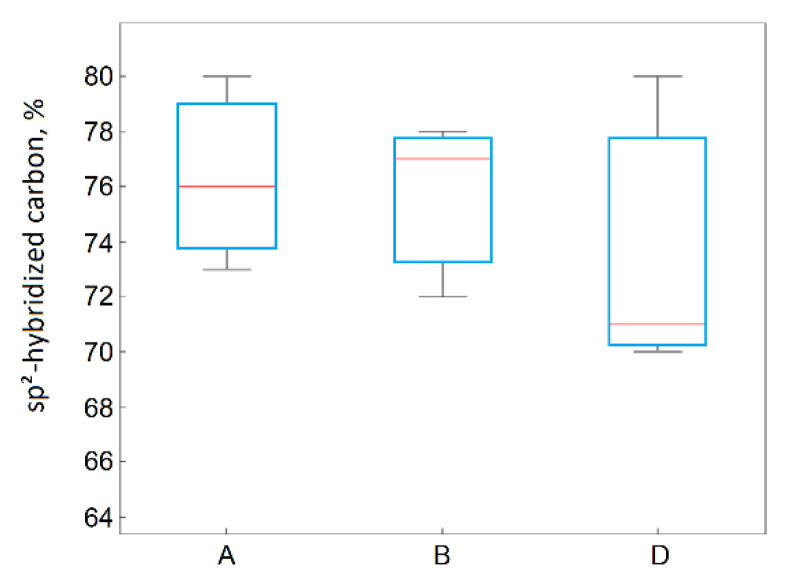
Box plot of C1sp^2^ hybridization as a function of the applied method.

In the process of thermal exfoliation–reduction, on the one hand, the content of carbon with sp^2^ hybridization increased, as shown in Figure 11. Due to the significant variance of the results in the case of the samples obtained using method D, it cannot be concluded that the differences in the carbon content with sp^2^ hybridization due to the applied method were statistically significant.

**Figure 12:**
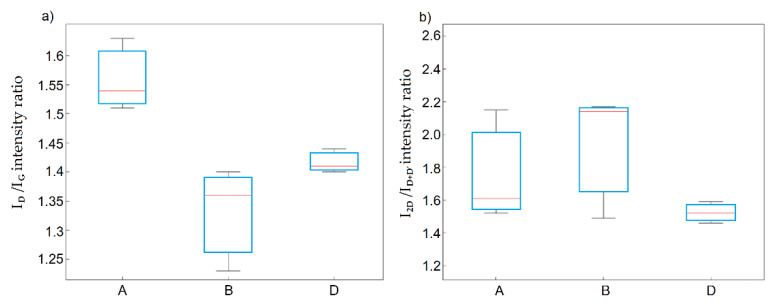
Box plots of (**a**) I_D_/I_G_ and (**b**) I_2D_/I_D+D’_ ratios as functions of the applied method.

On the other hand, the number of structural defects due to the elimination of oxygen groups increased. Based on the values of the I_D_/I_G_ and I_2D_/I_D+D’_ ratios determined from the Raman spectra, it was concluded that rGO obtained from the graphites oxidized using method A showed the highest amount of structural defects, while the lowest were for method B. The same observation was made for the values of interlayer distances d_00X_ from the XRD studies. The observed influence of the rGO method on the I_D_/I_G_ ratio was statistically significant and was confirmed by the ANOVA. For the I_2D_/I_D+D’_ ratio, this influence decreased, and due to the significant variability of the results, it became statistically insignificant (Figure 12a,b).

**Figure 13:**
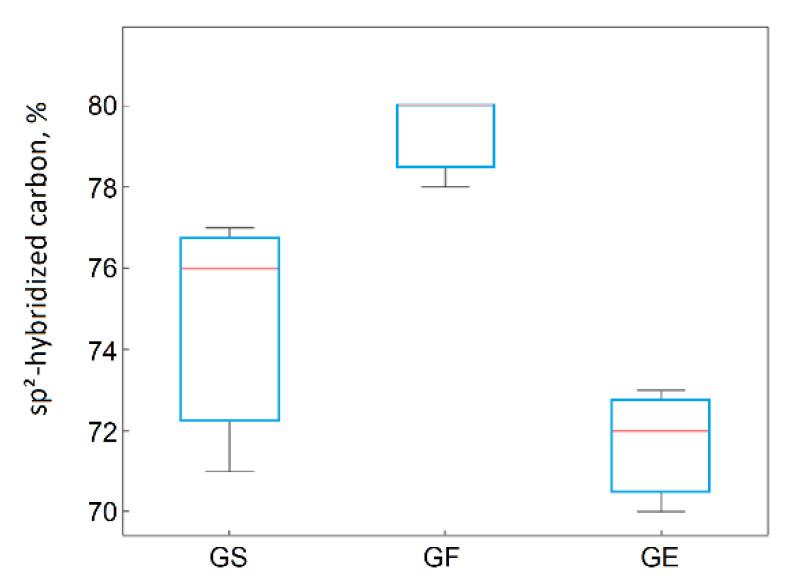
Box plot of C1sp^2^ hybridization as a function of the applied precursor material.

For materials obtained from flake graphite, as the graphite layers’ size in the graphite precursor increased, the I_D_/I_G_ ratio decreased and the structural defects in their structures also decreased. This was confirmed by the highest share of C sp^2^ being observed in the reduced graphene oxides obtained from flake graphite. The ANOVA also confirmed this observation. The precursor’s effect on the carbon content with C in sp^2^ hybridization was statistically significant (Figure 13).

## 4. Discussion

This statistical analysis allowed the following conclusions to be made for graphite oxide (GO):

- Based on the HCA method, it was determined that the graphite oxides with high and homogeneous oxygen contents, interlayer distances d_001_, and epoxy and hydroxyl group contents were obtained using methods A and D;
- Statistical analysis confirmed that the oxygen content in the graphite oxides was influenced by the oxidation method, while the graphite precursor had no significant influence on this;
- The ANOVA confirmed that the GOs obtained using method A contained the greatest interlayer distances d_001_ and the smallest dimensions for the L_a_ graphite layers;
- The ANOVA method showed the statistical significance of the influence of the graphite oxidation method on the degree of oxidation of GO. Method C, characterized by having the mildest oxidation conditions, resulted in GO samples with the least disturbed structure, and thus with the largest share of bonds with sp^2^ hybridization.

The influence of the graphite precursors (scaly graphite GS, flake graphite GF, and synthetic graphite GE) was statistically insignificant on the change in the I_D_/I_G_ ratio obtained for the graphite oxides. Research confirmed that method C had the weakest oxidizing potential.

Specific applications will require a GO with specific properties. The obtained results suggest the focus should be on the process variables of the graphite oxidation method, because this has a statistically significant impact on the prepared material’s properties.

This statistical study allowed the following conclusions to be made for the reduced graphene oxide (rGO):

- The used cluster analysis showed the most important variables, which characterize the degree of oxidation. It was confirmed with a high probability that an increase in the C(sp^2^) share in rGO indicates a partial reconstruction of the graphite structure;
- The statistical analysis showed the influence of the precursors (GS, GF) on the properties of the obtained reduced graphene oxides (the ANOVA analysis confirmed that the type of precursor influences the oxygen content and degree of reduction after thermal exfoliation–reduction). It is worth mentioning that there is also an influence on the I_D_/I_G_ ratio and number of structure defects;
- Moreover, the used ANOVA analysis showed statistically significant differences between the tested oxidation methods and the interlayer distance values d_00X_;
- The largest specific surfaces for rGO were obtained using method D. This type of reduced graphene oxide will be better suited for further technological processes for graphene production;
- On the other hand, rGO particles characterized by a higher degree of oxidation seem to be more appropriate for applications in gas sensors (especially in hydrogen sensors working in air atmosphere).

Apart from their cognitive value, the analyses presented above are essential for developing optimal technologies and may also have significant application value.

## 5. Conclusions

The results of our research and analyses using statistical methods confirmed the existence of relationships between the graphite precursor and used oxidation method related to the physicochemical properties obtained for GO and rGO.

In summary, the statistical evaluation of the results has allowed the development of preliminary criteria for selecting a graphite precursor for the production (using the thermal exfoliation–reduction method) of graphite material with the required texture and structural parameters. The obtained results suggest the choice of the production technology for reduced graphene oxide to obtain the desired graphene flakes.

The presented research suggests that after appropriate admixture or modification, the elaborated graphite oxide and reduced graphene oxide materials can be used in sensors of selected gases as sensing elements. The combination of reduced graphene oxides and other materials can increase the selected gas sensitivity and decrease the sensors’ operating temperature, as shown in our previous studies [24,33].

The utilitarian goal of the research presented in this paper is the use of reduced graphene oxide in hydrogen sensors with a fast reaction time (a few seconds), with great sensitivity, and working at room temperature.

## Figures and Tables

**Table 1:** The influence of the oxidation method on the oxygen content in the GO.

Oxidation Method	Material	Ratio C/O	[Ref.]
Brodie	natural graphite (Ceylon)	3.5	[3]
Staudenmaier	natural graphite	2.9	[4]
Hofmann	natural graphite	3.3	[5]
Hummers (various)	natural graphite with variable grain sizes smaller than 60 and at 60–90 µm	(16.0–2.0)	[6]
Hummers–Offemann	natural graphite	3.0	[7,8]

**Table 2:** GO preparation conditions [14,19].

Symbol	Base Acid	Oxidizing Mixture (Reagents)	Time
Method A	H_2_SO_4_ (20 mL)	HNO_3_ (15 mL), KMnO_4_ (3 g)	24 h
Method B	H_2_SO_4_ (30 mL)	NaNO_3_ (3 g), KMnO_4_ (3 g)	2 h
Method C	H_2_SO_4_ (22.5 mL)	NaNO_3_ (0.5 g), KMnO_4_ (3 g)	0.5 h
Method D	H_2_SO_4_ (45 mL), H_3_PO_4_ (5 mL),	KMnO_4_ (5 g), KNO_3_ (1.5 g)	5 h

## Supplementary Materials

The following are available online at https://www.mdpi.com/1996-1944/14/4/769/s1: Figure S1: The schematic presentation of normalization of all variables, Figure S2: Raman spectra of graphite oxides, Figure S3: Raman spectra of graphene oxides, Figure S4: XRD spectra of graphite oxides, Figure S5: XRD spectra of graphene oxides.

## References

[1] Singh V., Joung D., Zhai L., Das S., Khondaker S.I., Deal S. (2011). Graphene based materials: Past, present and future. Prog. Mater. Sci..

[2] Inagaki M., Kim Y.A., Endo M. (2011). Graphene: Preparation and structural perfection. J. Mater. Chem..

[3] Brodie B.C. (1960). Sur le poids atomique du graphite (On the atomic weight of graphite). Ann. Chim. Phys..

[4] Staudenmaier L. (1898). Verfahren zur darstellung der graphitsäure. Ber. Dtsch. Chem. Ges..

[5] Hofmann U., Holst R. (1939). Über die Säurenatur und die Methylierung von Graphitoxyd. Ber. Dtsch. Chem. Ges..

[6] Hummers W.S. (1954). Preparation of graphitic acid. U.S. Patent.

[7] Dimiev A.M., Eigler S. (2016). Graphene Oxide: Fundamentals and Applications.

[8] Chua C.K., Pumera M. (2014). Chemical reduction of graphene oxide: A synthetic chemistry viewpoint. Chem. Soc. Rev..

[9] Marcano D.C., Kosynkin D.V., Berlin J.M., Sinitskii A., Sun Z., Slesarev A., Alemany L.B., Lu W., Tour J.M. (2010). Improved synthesis of graphene oxide. ACS Nano.

[10] Sun L., Fugetsu B. (2013). Mass production of graphene oxide from expanded graphite. Mater. Lett..

[11] Botas C., Alvarez P., Blanco C., Santamarıa R., Granda M., Ares P., Rodriguez-Reinoso F., Menendez R. (2012). The effect of the parent graphite on the structure of graphene oxide. Carbon.

[12] Shao G., Lu Y., Wu F., Yang C., Zeng F., Wu Q. (2012). Graphene oxide: The mechanisms of oxidation and exfoliation. J. Mater. Sci..

[13] Luo D., Zhang G., Liu J., Sun X. (2011). Evaluation criteria for reduced graphene oxide. J. Phys. Chem..

[14] Muzyka R., Drewniak S., Pustelny T., Chrubasik M., Gryglewicz G. (2018). Characterization of graphite oxide and reduced graphene oxide obtained from different graphite precursors and oxidised by different methods using Raman spectroscopy. Materials.

[15] McAllister M.J., Li J.L., Adamson D.H., Schniepp H.C., Abdala A.A., Liu J., Herrera-Alonso M., Milius D.L., Car R., Prud’homme R.K. (2007). Single sheet functionalised graphene by oxidation and thermal expansion of graphite. Chem. Mater..

[16] Zhang C., Lv W., Xie X., Tang D., Liu C., Yang Q.-H. (2013). Towards low temperature thermal exfoliation of graphite oxide for graphene production. Carbon.

[17] Botas C., Perez-Mas A.M., Alvarez P., Santamaria R., Granda M., Blanco C., Menendez R. (2013). Optimization of the size and yield of graphene oxide sheets in the exfoliation step. Carbon.

[18] Deemer E.M., Paul P.K., Manciu F.S., Botez C.E., Hodges D.R., Landis Z., Akter T., Castro E., Chianelli R.R. (2017). Consequence of oxidation method on graphene oxide produced with different size graphite precursors. Mater. Sci. Eng. B.

[19] Muzyka R. (2018). Influence of Graphitic Precursor on the Composition, Morphology and Structure of Thermally Reduced Graphene Oxides. Ph.D. Thesis.

[20] Botas C., Alvarez P., Blanco C., Santamaria R., Granda M., Gutierrez M.D., Rodriguez-Reinoso F., Menendez R. (2013). Critical temperatures in the synthesis of graphene-like materials by thermal exfoliation-reduction of graphite oxide. Carbon.

[21] Schniepp H.C., Li J.L., McAllister M.J., Sai H., Herrera-Alonso M., Adamson D.H., Prud’homme R.K., Car R., Saville D.A., Aksay I.A. (2006). Functionalized single graphene sheets derived from splitting graphite oxide. J. Phys. Chem. B.

[22] Drewniak S.E., Pustelny T.P., Muzyka R., Plis A. (2015). Studies of physicochemical properties of graphite oxide and thermally exfoliated/reduced graphene oxide. Polish J. Chem. Technol..

[23] Drewniak S., Muzyka R., Drewniak Ł. (2020). The structure of thermally reduced graphene oxide. Photonics Lett. Poland.

[24] Drewniak S., Muzyka R., Stolarczyk A., Pustelny T., Kotyczka-Moranska M., Setkiewicz M. (2016). Studies of reduced graphene oxide and graphite oxide in the aspect of their possible application in gas sensors. Sensors.

[25] Davies M.P., De Biasi V., Perrett D. (2004). Approaches to the rational design of molecularly imprinted polymers. Anal. Chim. Acta.

[26] Navarro-Villoslada F., San Vicente B., Moreno-Bondi M.C. (2004). Application of multivariate analysis to the screening of molecularly imprinted polymers for bisphenol A. Anal. Chim. Acta.

[27] Potyrailo R.A. (2006). Polymeric sensor materials: Toward an alliance of combinatorial and rational design tools?. Angew. Chem. Int. Ed. Engl..

[28] Potyrailo R.A., Mirsky V.M. (2009). Combinatorial Methods for Chemical and Biological Sensors.

[29] Steinke J., Sherrington D.C., Dukin I.R. (1995). Imprinting of synthetic polymers using molecular templates. Adv. Polym. Sci..

[30] Abollino O., Aceto M., Malandrino M., Mentasti E., Sarzanini E., Barberis R. (2002). Distribution and mobility of metals in contaminated sites. Chemometric investigation of pollutant profiles. Environ. Pollut..

[31] Abollino O., Aceto M., Malandrino M., Mentasti E., Sarzanini C., Petrella F. (2002). Heavy metals in agricultural soils from Piedmont, Italy. Distribution, speciation and chemometric data treatment. Chemosphere.

[32] Sajdak M., Stelmach S., Kotyczka-Morańska M., Plis A. (2015). Application of chemometric methods to evaluate the origin of solid fuels subjected to thermal conversion. J. Anal. Appl. Pyrolysis.

[33] Pérez G., Valiente M. (2005). Determination of pollution trends in an abandoned mining site by application of a multivariate statistical analysis to heavy metals fractionation using SM&T–SES. J. Environ. Monitor..

