# Supplementary material for: Characterization of Graphite Oxide and Reduced Graphene Oxide Obtained from Different Graphite Precursors and Oxidized by Different Methods Using Raman Spectroscopy Statistical Analysis
Source: Materials (Basel). 2021 Feb 6;14(4):769. doi: 10.3390/ma14040769 (PMC7914510; doi:10.3390/ma14040769)
Supplement: Supplementary file 1 [file materials-14-00769-s001.pdf]

materials

Supplymentary Information

Characterization of Graphite Oxide and Reduced Graphene Oxide Obtained from Different Graphite Precursors and Oxidized by Different Methods Using Raman Spectroscopy Statistical Analysis

Roksana Muzyka [1], Sabina Drewniak [2,*], Tadeusz Pustelny [2], Marcin Sajdak [1] and Łukasz Drewniak [2]

[1] Institute for Chemical Processing of Coal, 1 Zamkowa St., 41–803 Zabrze, Poland; rmuzyka@ichpw.pl (R.M.); msajdak@ichpw.pl (M.S.)
[2] Department of Optoelectronics, Faculty of Electrical Engineering, Silesian University of Technology, 2 Krzywoustego St., 44–100 Gliwice, Poland; sabina.drewniak@polsl.pl (S.D.); tadeusz.pustelny@polsl.pl (T.P.); lukasz.drewniak@polsl.pl (L.D.)
* Correspondence: sabina.drewniak@polsl.pl

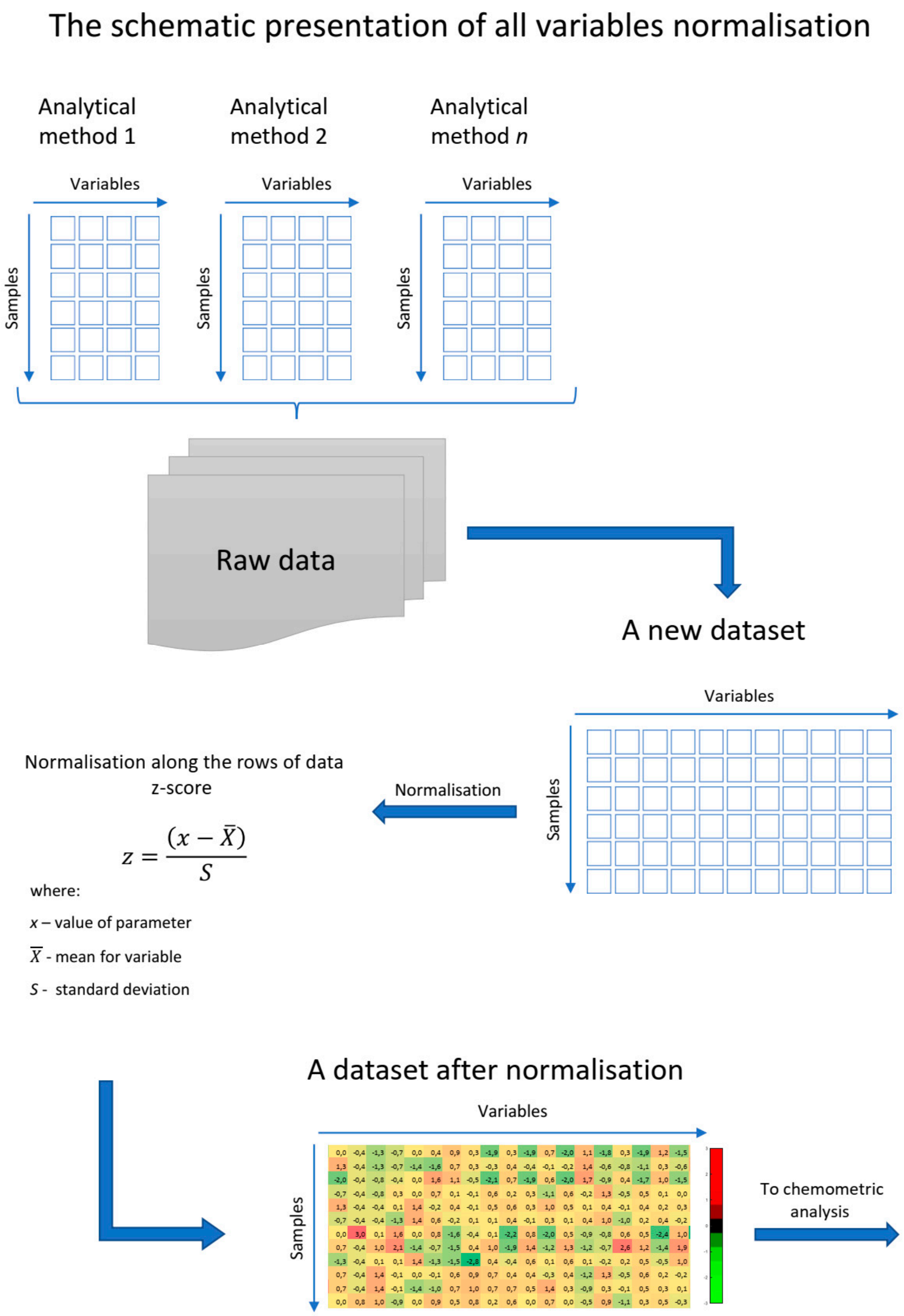

Figure S1. The schematic presentation of normalization of all variables.

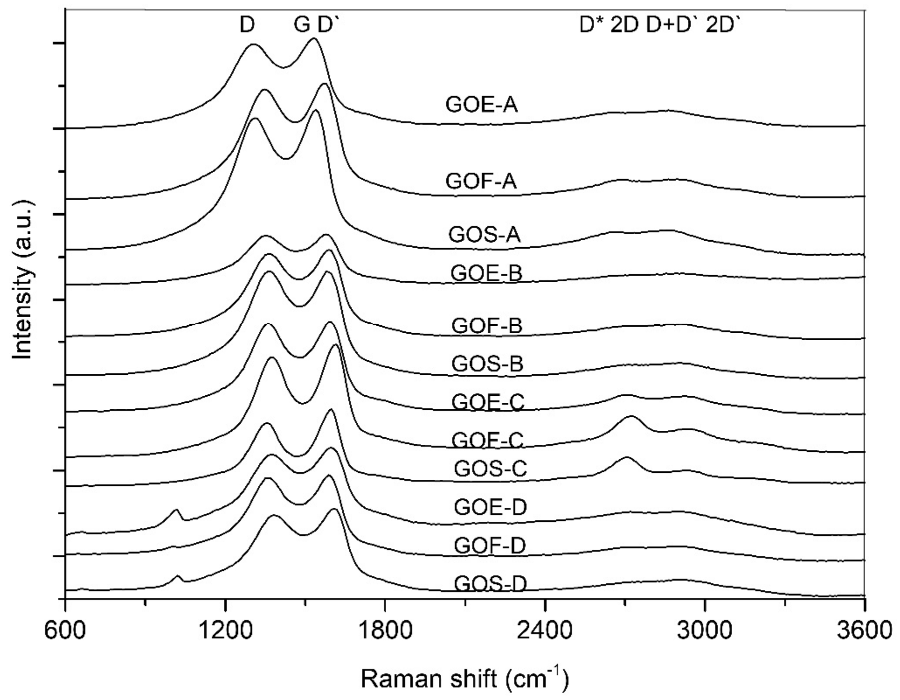

Figure S2. Raman spectra of graphite oxides.

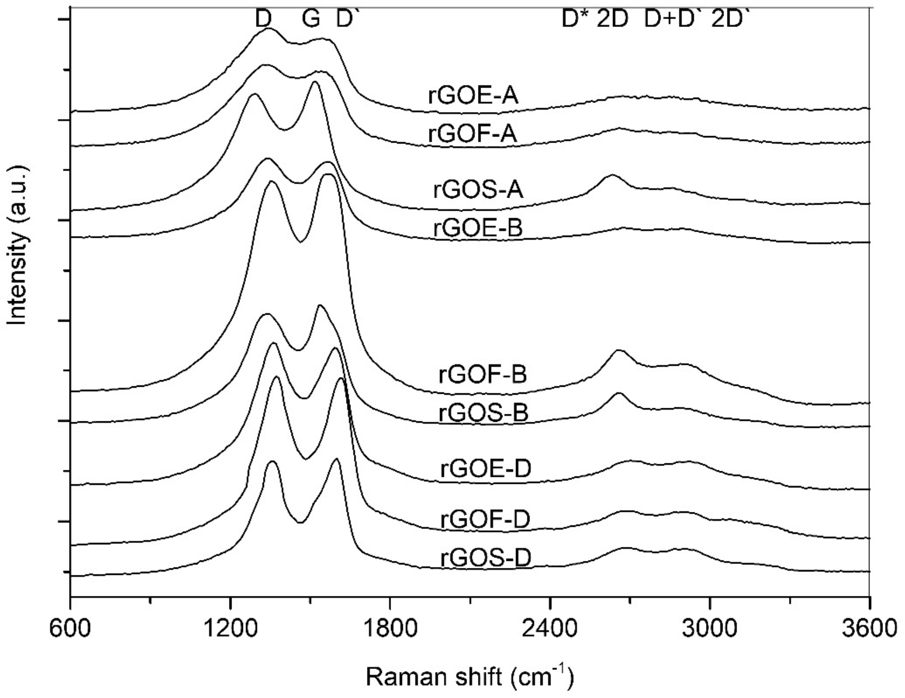

Figure S3. Raman spectra of reduced graphene oxides.

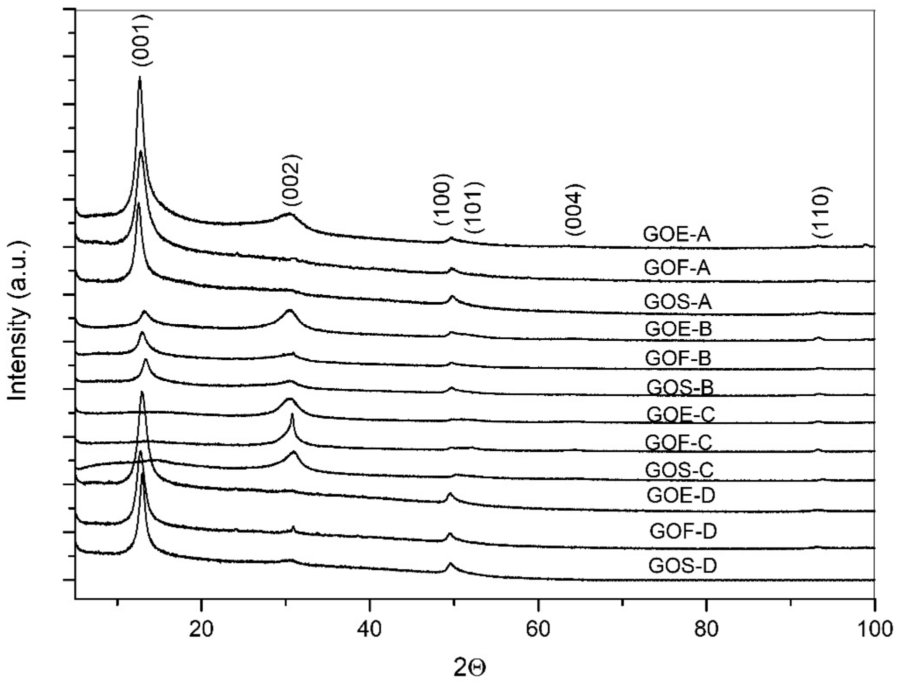

Figure S4. XRD spectra of graphite oxides.

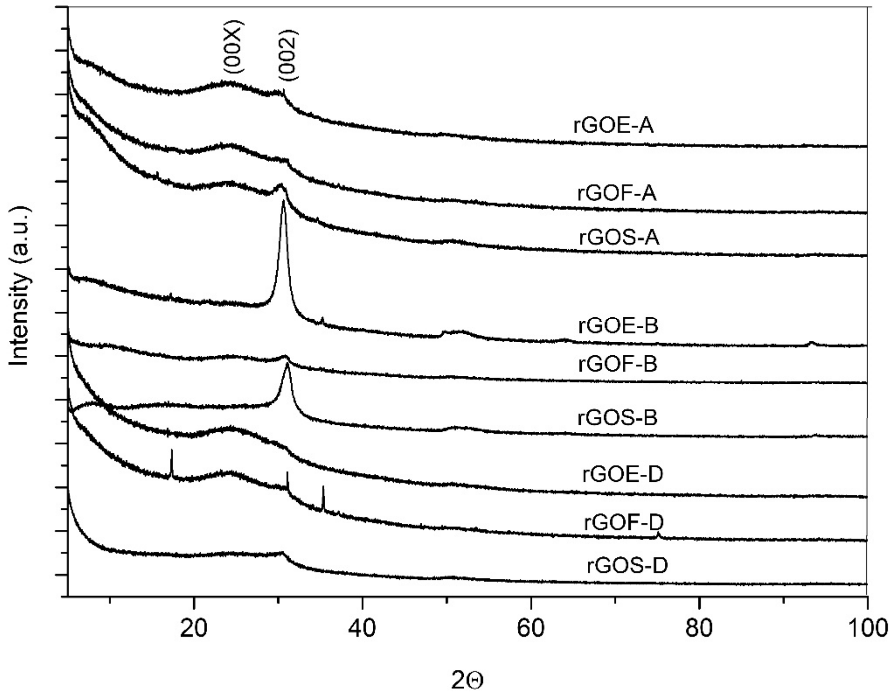

Figure S5. XRD spectra of reduced graphene oxides.
